# Skeletal responses to romosozumab after 12 months of denosumab

**DOI:** 10.1002/jbm4.10512

**Published:** 2021-06-03

**Authors:** Michael R. McClung, Michael A. Bolognese, Jacques P. Brown, Jean‐Yves Reginster, Bente L. Langdahl, Yifei Shi, Jen Timoshanko, Cesar Libanati, Arkadi Chines, Mary K. Oates

**Affiliations:** ^1^ Oregon Osteoporosis Center Portland Oregon USA; ^2^ Mary MacKillop Institute for Health Research Australian Catholic University Melbourne Victoria Australia; ^3^ Bethesda Health Research Center Bethesda Maryland USA; ^4^ Laval University and CHU de Quebec (CHUL) Research Centre Quebec City Quebec Canada; ^5^ University of Liege Liege Belgium; ^6^ King Saud University Riyadh Saudi Arabia; ^7^ Aarhus University Hospital Aarhus Denmark; ^8^ Amgen Inc. Thousand Oaks California USA; ^9^ UCB Pharma Brussels Belgium

**Keywords:** ANABOLIC, ANTIRESORPTIVE, DENOSUMAB, ROMOSOZUMAB, TREATMENT SEQUENCE

## Abstract

Romosozumab, a monoclonal anti‐sclerostin antibody that has the dual effect of increasing bone formation and decreasing bone resorption, reduces fracture risk within 12 months. In a post hoc, exploratory analysis, we evaluated the effects of romosozumab after 12 months of denosumab in postmenopausal women with low bone mass who had not received previous osteoporosis therapy. This phase 2 trial (NCT00896532) enrolled postmenopausal women with a lumbar spine, total hip, or femoral neck *T*‐score ≤ −2.0 and ≥ −3.5. Individuals were randomized to placebo or various romosozumab dosing regimens from baseline to month 24, were re‐randomized to 12 months of denosumab or placebo (months 24–36), and then all received romosozumab 210 mg monthly for 12 months (months 36–48). Results for the overall population have been previously published. Here, we present results for changes in bone mineral density (BMD) and levels of procollagen type I N‐terminal propeptide (P1NP) and β‐isomer of the C‐terminal telopeptide of type I collagen (β‐CTX) from a subset of women who were randomized to placebo for 24 months, were re‐randomized to receive denosumab (*n* = 16) or placebo (*n* = 12) for 12 months, and then received romosozumab for 12 months. In women who were randomized to placebo followed by denosumab, romosozumab treatment for 12 months maintained BMD gained during denosumab treatment at the total hip (mean change from end of denosumab treatment of 0.9%) and further increased BMD gains at the lumbar spine (mean change from end of denosumab treatment of 5.3%). Upon transition to romosozumab (months 36–48), P1NP and β‐CTX levels gradually returned to baseline from their reduced values during denosumab administration. Transitioning to romosozumab after 12 months of denosumab appears to improve lumbar spine BMD and maintain total hip BMD while possibly preventing the rapid increase in levels of bone turnover markers above baseline expected upon denosumab discontinuation. © 2021 The Authors. *JBMR Plus* published by Wiley Periodicals LLC on behalf of American Society for Bone and Mineral Research.

## Introduction

Antiresorptive drugs and bone‐forming agents are distinct classes of therapies for treating patients with osteoporosis.^(^ ^1^ ^)^ Estrogen receptor activators, bisphosphonates, and denosumab are anti‐remodeling drugs that reduce bone resorption and formation, increase bone mineral density (BMD), improve bone strength and reduce fracture risk, but they do not restore the disordered trabecular microarchitecture found in patients with postmenopausal osteoporosis.^(^ ^2^ ^)^ In contrast, bone‐forming agents stimulate bone formation, resulting in large increases in BMD and improved bone structure, and have been shown to be more effective at reducing fracture risk than oral bisphosphonates.^(^ ^3^, ^4^, ^5^, ^6^, ^7^ ^)^ Based on these data, bone forming agents are recommended as appropriate initial treatment for patients at very high risk of fracture.^(^ ^8^, ^9^, ^10^ ^)^

Romosozumab, an anti‐sclerostin monoclonal antibody, is a bone‐forming agent with a novel mechanism of action—a dual effect of activating both modeling‐based and remodeling‐based bone formation while reducing bone resorption.^(^ ^11^, ^12^ ^)^ In treatment‐naïve postmenopausal women with osteoporosis, romosozumab significantly improved bone mass and reduced fracture risk compared to placebo or alendronate.^(^ ^3^, ^13^, ^14^ ^)^ The benefits of the initial treatment with romosozumab for 12 months were maintained when patients were transitioned to either alendronate or denosumab.^(^ ^3^, ^13^, ^15^ ^)^ Some patients who have previously received antiresorptive therapies might also be candidates for romosozumab. Changes in BMD, estimated bone strength, and levels of serum bone turnover markers (BTMs) have been evaluated in patients transitioned from bisphosphonates to romosozumab.^(^ ^16^ ^)^ BMD and BTM responses to romosozumab in postmenopausal women who had taken denosumab for 12 months have also been reported;^(^ ^17^ ^)^ however, the women in that report had received romosozumab for 24 months before they received denosumab. To address a more common clinical scenario, we report here a post hoc, exploratory analysis of the effects of transitioning from denosumab to romosozumab in women with low bone mass not previously treated with romosozumab.

## Patients and Methods

### Study design and patients

**Fig. 1:**
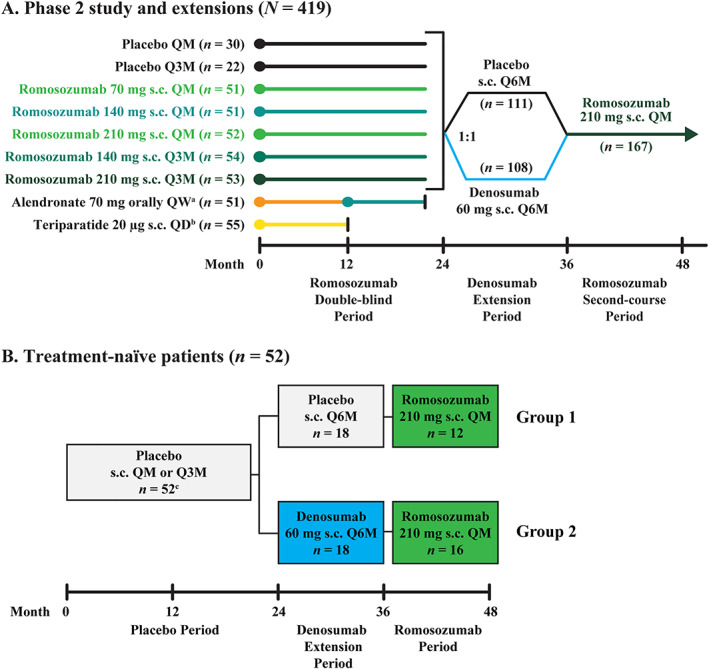
Phase 2 study design. (*A*) Women were randomized 1:1:1:1:1:1:1:1 to the first 24 months of treatment. Administration of placebo and the various romosozumab doses was blinded; alendronate and teriparatide were administered open‐label. At month 24, women were rerandomized (1:1) within treatment group to placebo or denosumab (60 mg s.c. Q6M) for 12 months, followed by a 12‐month second course of romosozumab 210 mg s.c. QM. (*B*) A subset of women who were randomized to receive placebo for 24 months (*n* = 52), rerandomized to receive denosumab (*n* = 16) or placebo (*n* = 12) for 12 months, and then received romosozumab for 12 months and whose results are presented in this report. ^a^Individuals transitioned to romosozumab 140 mg QM at month 12, were randomized in the denosumab extension period, completed the study at month 36 and are not included in the present analysis. ^b^Individuals completed the study at month 12 and are not included in the present analysis. ^c^Of the 52 women initially randomized to placebo from months 0 to 24, 18 were rerandomized to receive denosumab and 18 to receive placebo; the remaining 16 discontinued the study. Abbreviations: Q3M, every 3 months; Q6M, every 6 months; QD, daily; QM, monthly; QW, weekly; s.c., subcutaneously.

The women in this report were part of the romosozumab phase 2 dose‐finding study (NCT00896532; https://www.clinicaltrials.gov/ct2/show/NCT00896532). This international, multicenter, randomized, placebo‐controlled, parallel‐group study and its extensions had randomized 419 postmenopausal women 55 to 85 years old with low bone mass (*T*‐score of ≤ −2.0 and ≥ −3.5 at the lumbar spine, total hip, or femoral neck) into multiple arms and interventions; the details have been previously published.^(^ ^14^, ^17^, ^18^ ^)^ Briefly, women were randomized to receive one of five dosing regimens of subcutaneous romosozumab monthly or every 3 months (*n* = 261) or to receive one of two open‐label comparators (weekly oral alendronate 70 mg [*n* = 51] or daily subcutaneous teriparatide 20 μg [*n* = 55]) (Figure 1*A*; Supplemental Figure S1). The remaining 52 women were randomly assigned to receive subcutaneous placebo monthly or every 3 months. On completing the 12‐month double‐blind treatment period, women in the romosozumab and placebo groups continued their assigned treatment for an additional 12 months. At month 24, eligible consenting women entered a 12‐month extension phase and were re‐randomized (1:1) to double‐blind treatment with subcutaneous placebo or subcutaneous denosumab 60 mg every 6 months. Those who completed the month 24 to month 36 extension period then entered a 12‐month phase where they received open‐label subcutaneous romosozumab 210 mg monthly through month 48. All women were instructed to take calcium (≥1 g) and vitamin D (≥800 IU) daily throughout the study. In this analysis, we report the results from a subset of women who were randomized to receive placebo for 24 months, re‐randomized to receive denosumab or placebo for 12 months, and then received romosozumab for 12 months. This provides two treatment groups: one that received romosozumab after 3 years of placebo (Group 1; *n* = 12) and the second group that received placebo for 2 years, then denosumab for 12 months followed by 12 months of treatment with romosozumab (Group 2; *n* = 16) (Figure 1*B*). Only data from the 28 subjects who entered the month 36 to month 48 romosozumab treatment period are included in the analyses. One woman in Group 2, although assigned to receive placebo during months 0 to 12, received romosozumab during this period. To be consistent with data previously reported from this study, data for this woman was included in the efficacy but not the safety analyses.

### Study procedures

The study procedures for assessing BMD and BTMs have been previously published.^(^ ^14^, ^17^, ^18^ ^)^ Briefly, BMD at the lumbar spine and proximal femur were evaluated by dual‐energy x‐ray absorptiometry (DXA; Lunar, GE Medical Systems, Madison, WI, USA or Hologic, Hologic Inc., Bedford, MA, USA) at baseline (month 0) and months 3, 6, 12, 18, 24, 30, 36, 39, 42, and 48. BioClinica (previously known as Synarc; Newark, CA, USA) analyzed the scans and provided quality control of the individual scans and densitometers. Fasting serum samples were collected and analyzed to assess levels of procollagen type I N‐terminal propeptide (P1NP) (UniQ P1NP radioimmunoassay [RIA]; Orion Diagnostica Oy, Espoo, Finland) and β‐isomer of the C‐terminal telopeptide of type I collagen (β‐CTX) (Serum CrossLaps enzyme‐linked immunosorbent assay [ELISA]; Nordic Bioscience Diagnostics, A/S, Herlev, Denmark) at baseline through month 36 and then at months 37, 39, 42, 45, and 48.

### Study outcomes

Results for the whole population and for other treatment sequences have been previously published.^(^ ^14^, ^17^, ^18^, ^19^ ^)^ This report focuses on changes in BMD and P1NP and β‐CTX levels in a subset of women who received 12 months of romosozumab either after 36 months of placebo (Group 1) or after 24 months of placebo followed by 12 months of denosumab (Group 2).

A nonresponder analysis assessed the proportion of women with a BMD percentage decrease of >3% at the lumbar spine, total hip, or femoral neck during four time periods: baseline to 24 months, 24–36 months, 36–48 months, and 24–48 months. The 3% arbitrary cutoff for response versus nonresponse represents the approximate least significant change for the lumbar spine and total hip, as previously reported.^(^ ^15^ ^)^

### Statistical analysis

Data for all endpoints were summarized descriptively. Percentage changes from baseline in BMD are presented as means and 95% confidence intervals (CIs). Percentage changes from baseline in P1NP and β‐CTX are presented as medians and interquartile ranges. For the nonresponder analysis, number (*n*) and percentages of subjects are presented.

## Results

### Patients and baseline characteristics

Results of this phase 2 study and its extensions have been previously reported.^(^ ^14^, ^17^, ^18^ ^)^ This study reports data for months 24 to 48 for the 28 women who entered the month 36 to month 48 romosozumab treatment period (12 women in Group 1 who received romosozumab after 3 years of placebo; 16 women in Group 2 who received placebo for 2 years, then denosumab for 12 months followed by 12 months of treatment with romosozumab). No women in Group 1 discontinued the study while receiving romosozumab between month 36 and month 48. Two women in Group 2 discontinued the study during this period; 1 due to an adverse event and the other due to withdrawn consent.

Patient characteristics at baseline (month 0) and month 24 in the subset of women reported in this analysis were generally similar between groups (Table 1) with women in Group 2 being somewhat younger, having fewer years since menopause, and having higher levels of BTMs as compared to Group 1. Baseline characteristics for patients included in this analysis were also similar to those previously reported for the overall phase 2 study population.^(^ ^14^ ^)^

**Table 1:** Baseline characteristics of the subset of women who were randomized to placebo for 24 months, denosumab or placebo for 12 months, and then received romosozumab for 12 months

	Month 0 baseline		Month 24 baseline	
	Group 1 (*n* = 12)	Group 2 (*n* = 16)	Group 1 (*n* = 12)	Group 2 (*n* = 16)
Treatment from month 0–24	Placebo	Placebo	Placebo	Placebo
Treatment from month 24–36	Placebo	Denosumab 60 mg Q6M	Placebo	Denosumab 60 mg Q6M
Treatment from month 36–48	Romosozumab 210 mg QM	Romosozumab 210 mg QM	Romosozumab 210 mg QM	Romosozumab 210 mg QM
Age (years), mean ± SD	68.2 ± 6.5	63.8 ± 4.1	70.7 ± 6.4	66.1 ± 4.1
Years since menopause, mean ± SD	20.3 ± 9.8	16.9 ± 6.4	22.3 ± 9.8	18.9 ± 6.4
BMD *T*‐score, mean ± SD				
Lumbar spine	−2.3 ± 0.6	−2.4 ± 0.4	−2.1 ± 0.7	−2.4 ± 0.4
Total hip	−1.3 ± 0.7	−1.1 ± 0.6	−1.5 ± 0.7	−1.2 ± 0.6
Femoral neck	−1.8 ± 0.6	−1.6 ± 0.5	−1.9 ± 0.6	−1.7 ± 0.4
P1NP (μg/L), median (Q1, Q3)	37.0 (33.8, 41.0)	52.4 (44.9, 59.2)	38.2 (30.0, 55.6)	50.0 (40.0, 56.0)
β*‐*CTX (ng/L), median (Q1, Q3)	372.0 (306.0, 415.5)	503.5 (392.5, 635.5)	534.0 (433.5, 692.0)	626.0 (466.0, 833.0)

*Notes*: Reference ranges for the study are 9.7–92.5 μg/L for P1NP and 16.0–430.0 ng/L for β‐CTX. *n* = number of women enrolled from month 36 to month 48.

Abbreviations: β*‐*CTX, β‐isomer of the C‐terminal telopeptide of type I collagen; BMD, bone mineral density; P1NP, procollagen type I N‐terminal propeptide; Q1, first quartile; Q3, third quartile; Q6M, every 6 months; QM, monthly; SD, standard deviation.

### BMD

**Fig. 2:**
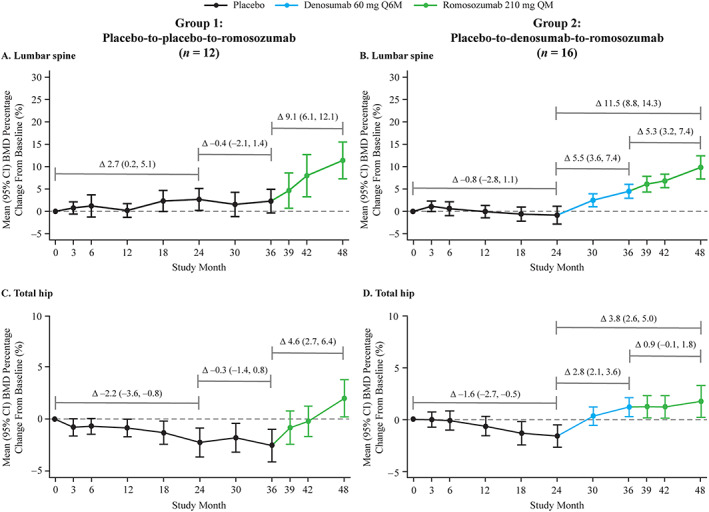
Percentage change from baseline in lumbar spine and total hip BMD through month 48 for (*A*,*C*) placebo‐to‐placebo‐to‐romosozumab and (*B*,*D*) placebo‐to‐denosumab‐to‐romosozumab. Data reported are for a subset of women who were randomized to receive placebo for 24 months (*n* = 52), rerandomized to receive denosumab or placebo for 12 months, and then received romosozumab for 12 months. *n* = number of women enrolled from month 36 to month 48. Abbreviations: BMD, bone mineral density; Q6M, every 6 months; QM, monthly.

BMD remained stable at the lumbar spine and decreased modestly in the proximal femur in women who received placebo for the first 24 months (Figure 2; Table 2). Increases in BMD at the lumbar spine, total hip, and femoral neck of 9.1%, 4.6%, and 3.9%, respectively, were observed during the 12‐month therapy (from month 24 to 36) with romosozumab in Group 1 (Figure 2*A*,*C*; Supplemental Figure S2*A*; Table 2). In Group 2, the average increase in BMD was 5.5%, 2.8%, and 2.3% at the lumbar spine, total hip, and femoral neck, respectively, during therapy with denosumab from month 24 to 36. In these women, lumbar spine BMD increased an additional 5.3% while taking romosozumab for 12 months for an average cumulative gain of 11.5% over the 24‐month denosumab‐to‐romosozumab treatment period (Figure 2*B*; Table 2). The increase in BMD seen with denosumab at the total hip was maintained by 12 months of romosozumab; 0.9% mean change from end of denosumab treatment, for a cumulative gain of 3.8% over the 24‐month treatment period with denosumab and romosozumab (Figure 2*D*; Table 2). Similarly, the femoral neck BMD increase noted with denosumab treatment was maintained by 12 months of romosozumab; 1.0% mean increase from end of denosumab treatment, for a cumulative gain of 3.2% over the last 2 years of the study (Supplemental Figure S2*B*; Table 2).

**Table 2:** Mean BMD percentage changes from baseline at the lumbar spine, total hip, and femoral neck

	Group 1 (*n* = 12)	Group 2 (*n* = 16)
Treatment from month 0–24	Placebo	Placebo
Treatment from month 24–36	Placebo	Denosumab 60 mg Q6M
Treatment from month 36–48	Romosozumab 210 mg QM	Romosozumab 210 mg QM
BMD (% change), mean (95% CI)		
Lumbar spine		
Month 0–24	2.7 (0.2, 5.1)	−0.8 (−2.8, 1.1)
Month 24–36	−0.4 (−2.1, 1.4)	5.5 (3.6, 7.4)
Month 36–48	9.1 (6.1, 12.1)	5.3 (3.2, 7.4)
Month 24–48	8.9 (5.5, 12.4)	11.5 (8.8, 14.3)
Total hip		
Month 0–24	−2.2 (−3.6, −0.8)	−1.6 (−2.7, −0.5)
Month 24–36	−0.3 (−1.4, 0.8)	2.8 (2.1, 3.6)
Month 36–48	4.6 (2.7, 6.4)	0.9 (−0.1, 1.8)
Month 24–48	4.7 (2.7, 6.7)	3.8 (2.6, 5.0)
Femoral neck		
Month 0–24	−1.3 (−2.7, 0.1)	−1.8 (−3.3, −0.4)
Month 24–36	−0.7 (−1.7, 0.3)	2.3 (1.0, 3.6)
Month 36–48	3.9 (1.7, 6.1)	1.0 (−1.0, 2.9)
Month 24–48	3.1 (0.8, 5.3)	3.2 (1.4, 5.0)

*Note*: *n* = number of women enrolled from month 36 to month 48.

Abbreviations: BMD, bone mineral density; CI, confidence interval; Q6M, every 6 months; QM, monthly.

The nonresponder analysis is presented in Table 3. None of the women who received romosozumab in Group 1 experienced a decrease in BMD of >3% at the lumbar spine, total hip, or femoral neck. Among the women in Group 2 who received denosumab from months 24 to 36, none had a decrease of >3% at the lumbar spine or total hip while receiving romosozumab during months 36–48. However, two of the 13 women in this group (15.4%) evidenced a decrease of femoral neck BMD of >3% (a decrease of 3.6% in one and 4.0% in another) while receiving romosozumab after 12 months of denosumab.

**Table 3:** Proportion of women who experienced BMD percentage decrease from baseline >3% at the lumbar spine, total hip, and femoral neck

	Group 1 (*n* = 12)	Group 2 (*n* = 16)
Treatment from month 0–24	Placebo	Placebo
Treatment from month 24–36	Placebo	Denosumab 60 mg Q6M
Treatment from month 36–48	Romosozumab 210 mg QM, *n2/n1* (%)	Romosozumab 210 mg QM, *n2/n1* (%)
Lumbar spine		
Month 0–24	0/12 (0)	7/16 (43.8)
Month 24–36	1/12 (8.3)	0/16 (0)
Month 36–48	0/11 (0)	0/13 (0)
Month 24–48	0/10 (0)	0/13 (0)
Total hip		
Month 0–24	5/12 (41.7)	4/16 (25.0)
Month 24–36	0/12 (0)	0/16 (0)
Month 36–48	0/11 (0)	0/13 (0)
Month 24–48	0/10 (0)	0/13 (0)
Femoral neck		
Month 0–24	3/12 (25.0)	5/16 (31.3)
Month 24–36	1/12 (8.3)	0/16 (0)
Month 36–48	0/11 (0)	2/13 (15.4)
Month 24–48	0/10 (0)	0/13 (0)

*Notes*: *n* = number of women enrolled from month 36 to month 48; *n1* = number of women with an evaluation; *n2* = number of women who experienced BMD percentage decrease from baseline >3%.

Abbreviations: BMD, bone mineral density; Q6M, every 6 months; QM, every month.

### BTMs

**Fig. 3:**
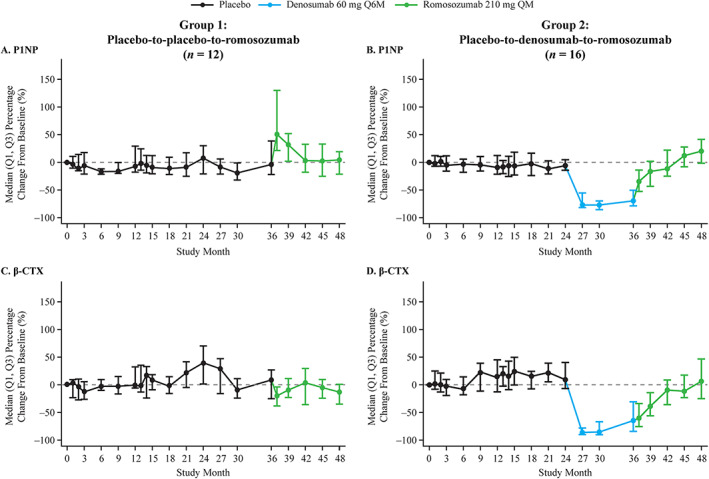
Percentage changes from baseline in serum P1NP and β‐CTX through month 48 for (*A*,*C*) placebo‐to‐placebo‐to‐romosozumab and (*B*,*D*) placebo‐to‐denosumab‐to‐romosozumab. Data reported are for a subset of women who were randomized to receive placebo for 24 months (*n* = 52), rerandomized to receive denosumab or placebo for 12 months, and then received romosozumab for 12 months. *n* = number of women enrolled from month 36 to month 48. Abbreviations: β‐CTX, β‐isomer of the C‐terminal telopeptide of type I collagen; P1NP, procollagen type I N‐terminal propeptide; Q1, first quartile; Q3, third quartile; Q6M, every 6 months; QM, monthly.

Median absolute levels of P1NP and β‐CTX are shown in Supplemental Table S1 and percentage changes from baseline are shown in Figure 3. While on placebo, P1NP and β‐CTX remained largely unchanged. In women who began romosozumab after 36 months of placebo, the observed patterns of BTM changes were similar to those reported^(^ ^13^, ^14^ ^)^ (Figure 3*A*,*C*). During 12 months of denosumab treatment, median serum levels of P1NP and β‐CTX decreased at all measured timepoints (months 24–36) (Figure 3*B*,*D*). Upon transition from denosumab to romosozumab (months 36–48), BTM levels gradually returned to levels just above baseline by month 48 for P1NP and to baseline by month 48 for β‐CTX (Figure 3*B*,*D*).

### Safety

Adverse events that started during months 36 to 48 are shown in Table 4. The adverse‐event profile during months 36 to 48 was similar to that in the first course of romosozumab 210 mg every month (month 0 to month 12^(^ ^14^ ^)^). Of the 28 women who entered the month 36 to month 48 romosozumab treatment period (12 in Group 1; 16 in Group 2), one woman in Group 2 had received romosozumab during months 0 to 12 of the double‐blind period and data for this woman was excluded from the safety analysis. Of the remaining 27 patients, adverse events were reported in 24 women; 12 (100.0%) in Group 1 and 12 (80.0%) in Group 2 experienced adverse events. A serious adverse event of invasive follicular thyroid carcinoma was reported in one (6.7%) patient in Group 2. Although not considered treatment related, the thyroid cancer led to discontinuation from the study. No fatal events were reported in either group.

**Table 4:** Subject incidence of adverse events during months 36 to 48

	Group 1 (*n* = 12)	Group 2 (*n* = 15)^a^
Treatment from month 0–24	Placebo	Placebo
Treatment from month 24–36	Placebo	Denosumab 60 mg Q6M
Treatment from month 36–48	Romosozumab 210 mg QM, *n1* (%)	Romosozumab 210 mg QM, *n1* (%)
All	12 (100.0)	12 (80.0)
Serious^b^	0 (0)	1 (6.7)
Leading to study discontinuation	0 (0)	1 (6.7)
Death	0 (0)	0 (0)
Adverse events of interest		
Potentially associated with hypersensitivity	1 (8.3)	1 (6.7)
Injection‐site reactions	0 (0)	2 (13.3)
Malignancies	0 (0)	1 (6.7)
Osteoarthritis	2 (16.7)	1 (6.7)
Atypical femoral fracture^c^	0 (0)	0 (0)
Hypocalcemia	0 (0)	0 (0)
Hyperostosis	0 (0)	0 (0)
Osteonecrosis of the jaw^c^	0 (0)	0 (0)

*Notes*: Only adverse events starting during months 36 to 48 are included. *n* = number of participants in each treatment group who received at least one dose of romosozumab during months 36 to 48; *n1* = number of participants reporting at least one adverse event.

Abbreviations: Q6M, every 6 months; QM, every month.

^a^ Of the 16 women in Group 2, one had received romosozumab during months 0 to 12 of the double‐blind period; data for this woman was excluded from the safety analysis.

^b^ One woman in Group 2 had invasive follicular thyroid carcinoma deemed by the investigator not to be related to the study drug; the woman was discontinued from the drug and study due to the cancer.

^c^ All potential events of osteonecrosis of the jaw and atypical femur fracture from the start of the study were retrospectively assessed for adjudication.

Adverse events potentially associated with hypersensitivity were reported in two women, one in Group 1 and the other in Group 2. Injection‐site reactions, mostly mild in severity, were reported in two (13.3%) women in Group 2 and malignancy was reported in one (6.7%) woman in Group 2. Osteoarthritis was reported in three women; two (16.7%) women in Group 1 and one (6.7%) woman in Group 2. There were no reports of hyperostosis, hypocalcemia, positively adjudicated osteonecrosis of the jaw, or positively adjudicated atypical femur fracture.

## Discussion

In this exploratory analysis, transitioning from 12 months of denosumab to romosozumab for 12 months resulted in a gradual increase in serum markers of bone formation and resorption to or slightly above baseline levels from the low values observed while on denosumab. The BMD gain achieved with denosumab was generally maintained at the proximal femur and increased by an additional 5.3% at the lumbar spine.

Some aspects of these results are notable. First, neither the rapid increase in levels of BTMs to values above baseline nor the rapid decrease in BMD that accompanies denosumab discontinuation was observed when therapy was switched from denosumab to romosozumab.^(^ ^20^, ^21^ ^)^ These results differ from those seen upon transitioning from denosumab to teriparatide where BTMs increased well above baseline, and BMD in the proximal femur declined significantly during the first 12 months of teriparatide therapy.^(^ ^22^ ^)^ Second, the gains in BMD with 12 months of romosozumab in the group that had previously received 12 months of denosumab were smaller than those achieved over 12 months when romosozumab was the first therapy. Smaller increments in BMD were also observed with romosozumab therapy in postmenopausal women who had previously received bisphosphonate therapy compared with responses typically observed in those who had not taken osteoporosis medications.^(^ ^14^, ^16^ ^)^ Moreover, the total gain in total hip and femoral neck BMD achieved with 12 months of denosumab followed by 12 months of romosozumab in this study was smaller than that achieved with 12 months of romosozumab in women who had previously received only placebo. Finally, increases in BMD over the 24‐month sequence of denosumab followed by romosozumab were smaller (lumbar spine +11.5%, total hip +3.8%, femoral neck +3.2%) than the reported changes in women with osteoporosis who received 12 months of romosozumab followed by denosumab for an additional 12 months in the Fracture Study in Postmenopausal Women with Osteoporosis (FRAME) (lumbar spine +16.6%, total hip +8.5%, femoral neck +7.3%).^(^ ^13^ ^)^

The observed differences in BMD responses depending upon the sequence with which romosozumab and denosumab are given have important clinical implications. Recent studies have shown associations between current fracture risk and proximal femur BMD achieved on treatment with alendronate, denosumab, or romosozumab.^(^ ^23^, ^24^ ^)^ These observations are supported by the meta‐regression analysis showing that therapies that produce the largest increases in BMD are associated with the greatest reductions in fracture risk.^(^ ^25^ ^)^ Collectively, these results support the use of a bone‐forming agent first followed by a potent antiresorptive drug as an optimal treatment sequence, particularly for women at very high risk of fracture.^(^ ^15^, ^26^ ^)^

The smaller BMD gains observed with romosozumab following denosumab are consistent with other studies showing that changes in BMD both with antiresorptive drugs and teriparatide are related to baseline levels of bone turnover.^(^ ^27^, ^28^, ^29^ ^)^ BTM levels are routinely lower in women receiving denosumab compared with pretreatment values.^(^ ^30^, ^31^ ^)^ Our results are also consistent with a recent observational study demonstrating smaller BMD increases with romosozumab in women who had received previous antiresorptive therapy.^(^ ^32^ ^)^

Interpreting the BMD and BTM responses in women transitioning from denosumab to any other therapy is complicated by the dynamic changes in bone remodeling known to occur in the several months following denosumab discontinuation.^(^ ^20^, ^21^ ^)^ The BMD response during the 12 months of romosozumab after denosumab in our study is, at least in relative terms, greater than is apparent because a significant decrease in bone density would have been expected upon stopping denosumab without further therapy.^(^ ^20^, ^21^ ^)^

The clinical value of the present study is that this is the first study to evaluate the sequence of denosumab followed by romosozumab in treatment‐naïve postmenopausal women. Important limitations to our study need to be acknowledged. The small number of individuals and the post hoc, exploratory nature of our study precludes firm conclusions about the effectiveness of romosozumab to prevent the rebound in bone remodeling and loss of bone density upon discontinuation of denosumab, and our results should be interpreted with caution. Additionally, the short duration of denosumab therapy (12 months) before transitioning to romosozumab might not reflect responses to that of transition after longer exposure to denosumab. Furthermore, the women in our study had low bone mass and were not at high risk of fracture and thus might not be representative of subjects most likely to receive romosozumab therapy.

In conclusion, romosozumab appears to maintain or improve the gains in BMD after a 12‐month course of denosumab treatment in postmenopausal women with low bone mass, while levels of BTMs gradually return to baseline levels. The sequence of romosozumab after denosumab is not as effective in increasing BMD as is the opposite sequence of using romosozumab first. Larger studies in women at high fracture risk are needed to determine the true clinical impact and utility of the treatment sequence of denosumab followed by romosozumab.

## Disclosures

Michael R. McClung has received consulting fees from Amgen and Myovant and has received honorarium from Amgen and Alexion. Michael A. Bolognese has received contract fees and speaker fees from Amgen. Jacques P. Brown has received grants/research support from Mereo BioPharma, Radius Health, and Servier; has received consulting fees from Amgen and Servier; and has served on a speakers' bureau for Amgen. Jean‐Yves Reginster has received grants/research support from IBSA‐Genevrier, Mylan, CNIEL, and Radius Health; has received lecture fees from IBSA‐Genevrier, Mylan, CNIEL, and Dairy Research Council; and has received consulting fees/participated on advisory boards for IBSA‐Genevrier, Mylan, Radius Health, and Pierre Fabre. Bente L. Langdahl has received grants/research support from Amgen and Novo Nordisk and has served on speakers' bureaus for UCB Pharma, Amgen, Eli Lilly, Gedeon‐Richter, and Gilead. Yifei Shi, Arkadi Chines, and Mary K. Oates are employees of and own stock in Amgen. Jen Timoshanko and Cesar Libanati are employees of and own stock in UCB Pharma.

## Author Contributions

The first author (Michael R. McClung) and three authors from Amgen Inc. (Yifei Shi, Arkadi Chines, and Mary K. Oates) take responsibility for the integrity of the data analysis. The first author (Michael R. McClung) wrote the first draft of the manuscript. All authors participated in the analysis and/or interpretation of the data, and participated in the critical review and revision of subsequent draft of the manuscript. All authors approved the final version of the manuscript and agreed to submit it to *JBMR Plus* for publication.

## Data Sharing

Qualified researchers may request data from Amgen clinical studies. Complete details are available: https://wwwext.amgen.com/science/clinical-trials/clinical-data-transparency-practices/clinical-trial-data-sharing-request/.

## Supporting information


**Appendix S1**: Supporting Information
