# Supplementary material for: Skeletal responses to romosozumab after 12 months of denosumab
Source: JBMR Plus. 2021 Jun 3;5(7):e10512. doi: 10.1002/jbm4.10512 (PMC8260819; doi:10.1002/jbm4.10512)
Supplement: Supplementary file 1 — Appendix S1: Supporting Information [file JBM4-5-e10512-s001.pdf]

Electronic Supplemental Material

Skeletal Responses to Romosozumab after 12 Months of Denosumab

Michael R. McClung, MD;[1] Michael A. Bolognese, MD;[2] Jacques P. Brown, MD;[3] Jean-Yves Reginster, MD, PhD;[4] Bente L. Langdahl, MD, PhD;[5] Yifei Shi, MS;[6] Jen Timoshanko, PhD;[7] Cesar Libanati, MD;[7] Arkadi Chines, MD;[6] Mary K. Oates, MD[6]

[1]Oregon Osteoporosis Center, Portland, OR, USA and Mary MacKillop Institute for Health Research, Australian Catholic University, Melbourne, VIC, Australia; [2]Bethesda Health Research Center, Bethesda, MD, USA; [3]Laval University and CHU de Quebec (CHUL) Research Centre, Quebec City, QC, Canada; [4]University of Liege, Liege, Belgium and King Saud University, Riyadh, Kingdom of Saudi Arabia; [5]Aarhus University Hospital, Aarhus, Denmark; [6]Amgen Inc., Thousand Oaks, CA, USA; [7]UCB Pharma, Brussels, Belgium

Funding

This study was funded by Amgen Inc., UCB Pharma, and Astellas Pharma Inc.

Corresponding author

Michael R. McClung, MD; Oregon Osteoporosis Center, 2881 NW Cumberland Road, Portland, OR 97210, USA. Phone: + 1 503-929-9633. Email: mmcclung.ooc@gmail.com

Journal: *JBMR Plus*
Electronic supplemental material: 1 table and 2 figures

Supplemental Figure 1. Disposition of all study women through Month 48

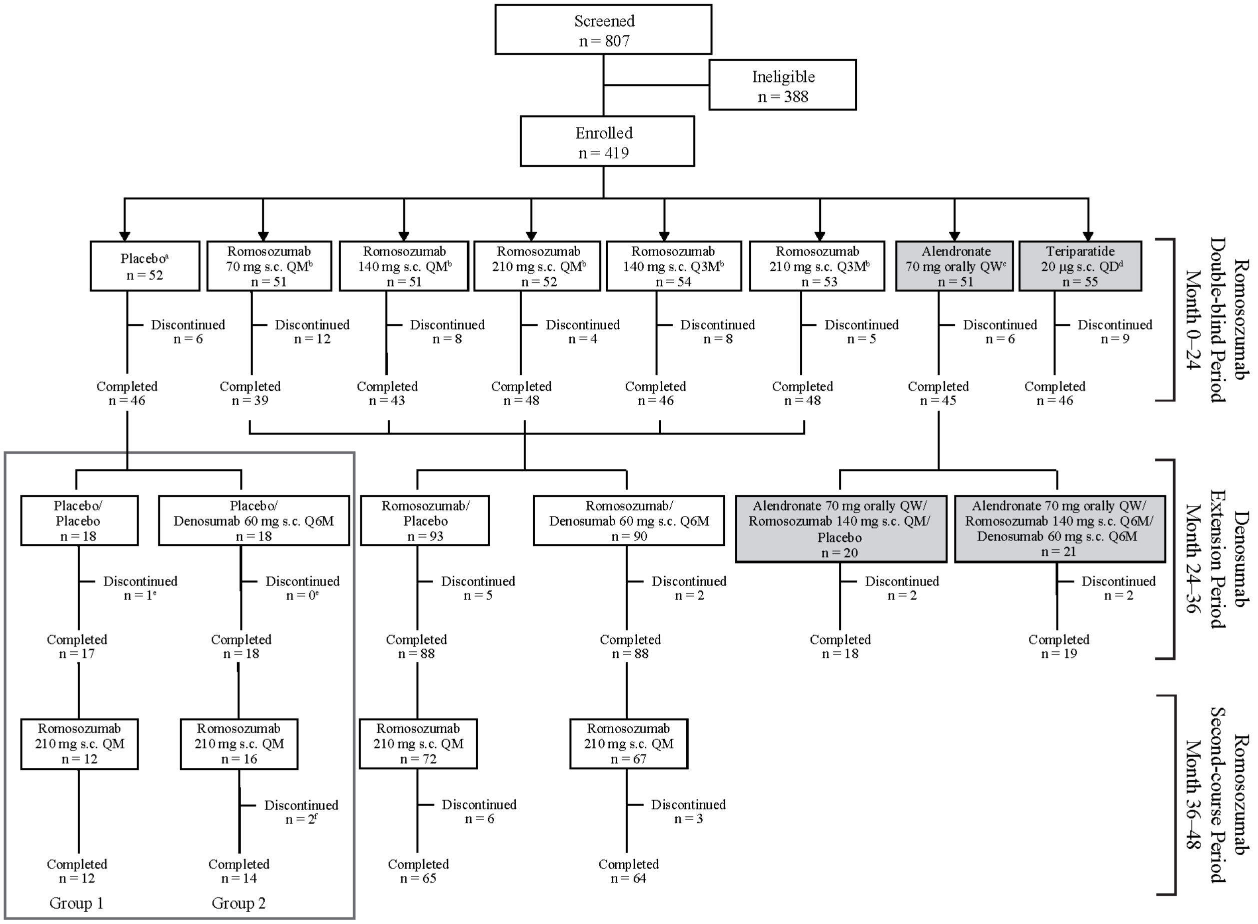

[a]At Month 12, women initially randomized to receive placebo continued to receive placebo up to Month 24.
[b]At Month 12, women initially randomized to receive a specific dose and schedule of romosozumab continued to receive their assigned treatment up to Month 24.
[c]At Month 12, women initially randomized to receive alendronate (*gray box*) were transitioned to receive romosozumab 140 mg QM for 12 months up to Month 24, were randomized to the denosumab extension period, and completed the study at Month 36 and are not included in the present analysis.
[d]Women initially randomized to receive teriparatide (*gray box*) completed the study at Month 12 and are not included in the present analysis.
[e]One woman in Group 1 discontinued the study between Month 24 and Month 36 due to protocol deviation; none of the women in Group 2 discontinued the study during this period.
[f]No women in Group 1 discontinued the study between Month 36 and Month 48; 2 women in Group 2 discontinued the study during this period; one due to an adverse event and the other due to consent withdrawal.
QD = daily; QM = monthly; Q3M = every 3 months; Q6M = every 6 months; QW = weekly.

Supplemental Figure 2. Percentage change from baseline in femoral neck BMD through Month 48 for **A**) placebo-to-placebo-to-romosozumab and **B**) placebo-to-denosumab-to-romosozumab.

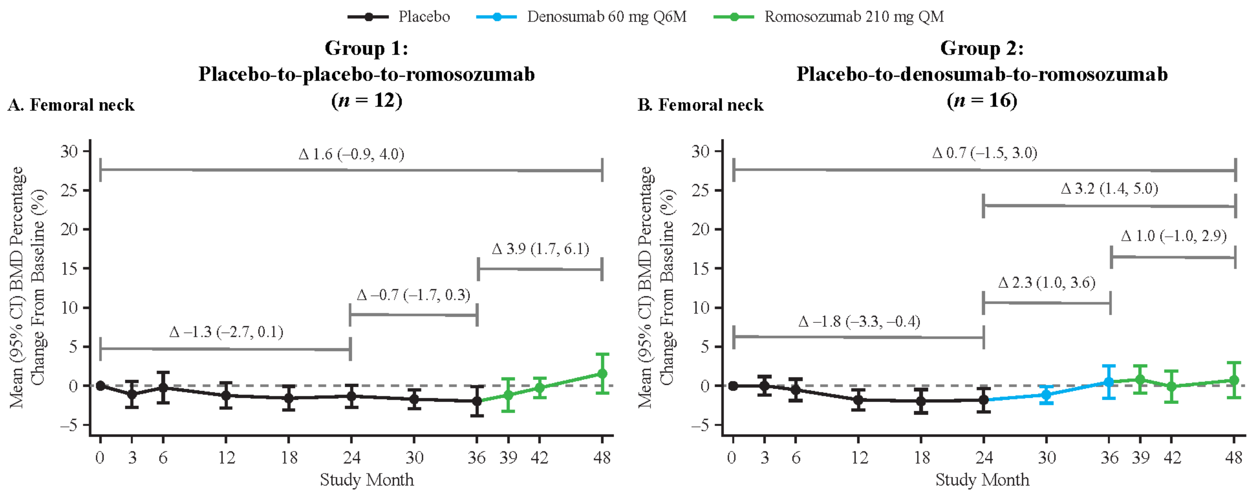

Data reported are for a subset of women who were randomized to receive placebo for 24 months (n = 52), rerandomized to receive denosumab or placebo for 12 months, and then received romosozumab for 12 months. n = number of women enrolled from Month 36 to Month 48. QM = monthly; Q6M = every 6 months.

Supplemental Table 1. Median Absolute Levels of P1NP and β-CTX from Month 0 to Month 48

Treatment from Month 0–24:<br>Treatment from Month 24–36:<br>Treatment from Month 36–48:	Group 1<br>Placebo<br>Placebo<br>Romosozumab 210 mg QM<br>*n* = 12	Group 2<br>Placebo<br>Denosumab 60 mg Q6M<br>Romosozumab 210 mg QM<br>*n* = 16
P1NP, median (Q1, Q3), μg/L		
Baseline	37.0 (33.8, 41.0)	52.4 (44.9, 59.2)
Day 1 + 1 Week	37.1 (31.8, 39.7)	52.2 (40.2, 63.4)
Month 1	34.1 (31.3, 44.7)	55.5 (48.3, 64.5)
Month 2	35.2 (32.5, 38.8)	57.9 (47.3, 60.4)
Month 3	33.5 (31.9, 46.9)	50.6 (43.2, 62.8)
Month 6	31.2 (27.9, 45.5)	52.0 (39.3, 61.3)
Month 9	31.3 (29.4, 43.8)	49.6 (39.0, 63.8)
Month 12	36.3 (32.4, 52.8)	47.4 (40.8, 61.0)
Month 12 + 1 Week	41.1 (34.0, 49.3)	47.9 (39.7, 61.1)
Month 13	36.6 (33.7, 47.1)	45.3 (41.3, 54.5)
Month 14	34.0 (29.8, 49.9)	53.9 (38.0, 62.3)
Month 15	35.7 (31.1, 40.7)	51.6 (39.7, 57.9)
Month 18	33.0 (30.3, 42.6)	52.8 (37.8, 64.7)
Month 21	34.5 (26.1, 46.4)	45.7 (35.6, 53.5)
Month 24	38.2 (30.0, 55.6)	50.0 (40.0, 56.0)
Month 27	32.5 (28.4, 42.8)	11.4 (10.2, 20.3)
Month 30	28.9 (26.5, 44.2)	10.8 (8.1, 13.4)
Month 36	35.9 (30.3, 55.5)	17.4 (11.2, 21.4)
Month 37	50.6 (47.8, 107.7)	36.6 (22.3, 44.9)
Month 39	49.5 (36.3, 79.9)	43.2 (31.6, 55.6)
Month 42	42.0 (31.3, 55.9)	50.2 (43.4, 64.5)
Month 45	40.9 (27.4, 48.1)	61.6 (53.2, 74.4)
Month 48	36.2 (29.2, 48.2)	64.6 (54.3, 72.5)
β-CTX, median (Q1, Q3), ng/L		
Baseline	372 (306, 416)	504 (393, 636)
Day 1 + 1 Week	369 (260, 416)	466 (422, 608)
Month 1	363 (280, 416)	505 (438, 695)
Month 2	341 (269, 458)	492 (431, 659)
Month 3	348 (270, 442)	501 (417, 570)
Month 6	354 (308, 448)	452 (377, 584)
Month 9	363 (278, 443)	558 (463, 699)
Month 12	413 (313, 594)	485 (422, 752)
Month 12 + 1 Week	444 (334, 541)	601 (470, 775)
Month 13	373 (278, 558)	493 (434, 693)
Month 14	458 (272, 494)	568 (413, 770)
Month 15	414 (322, 492)	572 (465, 813)
Month 18	350 (313, 596)	562 (375, 671)
Month 21	467 (346, 556)	613 (465, 827)

	Group 1	Group 2
Treatment from Month 0–24:	Placebo	Placebo
Treatment from Month 24–36:	Placebo	Denosumab 60 mg Q6M
Treatment from Month 36–48:	Romosozumab 210 mg QM	Romosozumab 210 mg QM
	n = 12	*n* = 16
Month 24	534 (434, 692)	626 (466, 833)
Month 27	494 (308, 680)	52 (52, 103)
Month 30	320 (266, 448)	71 (52, 135)
Month 36	376 (305, 534)	163 (96, 268)
Month 37	306 (261, 371)	182 (135, 248)
Month 39	348 (282, 439)	311 (239, 385)
Month 42	348 (318, 490)	446 (343, 577))
Month 45	355 (299, 422)	495 (348, 710)
Month 48	321 (277, 407)	532 (378, 661)

Reference ranges for the study are 9.7–92.5 μg/L for P1NP and 16.0–430.0 ng/L for β-CTX.
n = number of women enrolled from Month 36 to Month 48.
Q1, Q3 = first and third quartiles; QM = monthly; Q6M = every 6 months.
